# Reply to Comment on Detection of Mycotoxins in Patients with Chronic Fatigue Syndrome *Toxins* 2013, *5*, 605–617 by John W. Osterman, M.D. †

**DOI:** 10.3390/toxins8110323

**Published:** 2016-11-07

**Authors:** Joseph Brewer, Jack Dwayne Thrasher, Dennis Hooper

**Affiliations:** 1 Plaza Infectious Disease and St. Luke’s Hospital, 4320 Wornall Road, Suite 440, Kansas City, MO 64111, USA; 2 Citrus Heights, CA 95610, USA; toxicologist1@msn.com; 3 RealTime Laboratories, Carrollton, TX 75010, USA; dhooper@realtimelab.com

This paper [1] was an observational case study. It was not intended to be, nor have we ever indicated that it was, an epidemiologic study [2]. One of the authors (Dr. Brewer) is an infectious disease specialist, who treats a number of patients with chronic fatigue syndrome (CFS). Dr. Brewer’s primary responsibility is to properly diagnose and treat these patients and ensure their wellbeing. In 2012, Dr. Brewer began to test patients for the presence of mycotoxins using the RealTime Lab’s mycotoxin panel. As he saw and treated more and more chronic fatigue patients, he began to see an association between the presence of mycotoxins and the symptoms of CFS. As this association became more apparent, Dr. Brewer discussed these findings with other experts in the field of mycotoxins. It was decided that these observations had potentially important clinical implications and the group decided to proceed with publication of this collection of clinical cases. The patients reported in our study were included based on their diagnosis (CFS) and not their exposure history.

These observations did lead to a hypothesis that perhaps the patients had internal fungal growth leading to both the symptoms of CFS and the presence of the mycotoxins produced by the fungi. Subsequently, this resulted in a treatment regimen for fungal colonization/infection in the sinuses, the results of which improved both the patient’s health and reduced the concentration of mycotoxins.

Never did the authors state or imply that mycotoxins caused CFS and never did we undertake a controlled study to look at CFS in a mycotoxin positive and a mycotoxin negative population. The major finding was the association between mycotoxins and CFS. In the paper (discussion section) several ideas were addressed (e.g., mitochondrial toxicity) as to possible pathophysiologic mechanisms.

The reference to the negative controls of another study, where the individuals were not exposed to a water damaged and potentially mold infested environment, was only meant to point out that the entire general population does not harbor elevated levels of mycotoxins, and/or the molds that produce them (despite low levels of exposure in the environment and potential mycotoxin-exposure in foods).

Much work would be and is needed to link mycotoxins and or mold as the causative agent of CFS and the authors understand that this would necessitate a clinical study with the appropriate mycotoxin negative controls. While this may be a future project, the focus now is on patient treatment and presentation of case histories such as the ones in this paper.

In summary, this was a clinical observation, not an epidemiological study. The findings are provocative and may have important implications for these types of illnesses. The results will hopefully stimulate and promote further investigation by our group and others.
